# Can transcutaneous bilirubin levels obtained from covered skin replace serum bilirubin measurement in neonates undergoing phototherapy?

**DOI:** 10.2478/abm-2025-0025

**Published:** 2025-09-02

**Authors:** M. Fatih Ozden, Dilek Kahvecioglu, Melda Tas, Aslihan Kose Cetinkaya, Ahmet Oktem

**Affiliations:** Department of Pediatrics, University of Health Sciences Ankara Training and Research Hospital, Ankara 06230, Turkey; Department of Neonatology, University of Health Sciences Ankara Training and Research Hospital, Ankara 06230, Turkey

**Keywords:** bilirubin, neonatal jaundice, newborn, non-invasive monitoring, phototherapy, transcutaneous bilirubinometry

## Abstract

**Background:**

Transcutaneous bilirubinometers provide a non-invasive method to estimate total serum bilirubin (TSB) using multiwavelength reflectance. However, their accuracy during phototherapy (PT) remains controversial due to decreased dermal bilirubin.

**Objective:**

To assess the correlation between TSB and transcutaneous bilirubin (TcB) measured from covered and exposed skin areas before, during, and after PT.

**Methods:**

In this prospective study, 70 neonates undergoing PT were assessed. TcB measurements were obtained from the exposed sternum and the forehead, which were covered with an aluminum-coated radiopaque patch. TSB and TcB values were measured before PT, at 24 h during treatment, and 8 h post-treatment. The agreement between TSB and TcB values was evaluated using the intraclass correlation coefficient (ICC).

**Results:**

TcB values of covered skin showed significant correlation with TSB at all-time points (ICC = 0.665 for pre-PT, ICC = 0.520 at 24 h, and ICC = 0.537 for post-treatment). TcB values of exposed skin showed poor correlation during and after PT. Mean differences between covered TcB and TSB remained within acceptable limits (<1 mg/dL).

**Conclusion:**

TcB measurements from covered skin offer a reliable, non-invasive alternative to serum bilirubin levels in neonates receiving PT, reducing the need for repeated blood draws.

Jaundice is a physiological condition caused by hyperbilirubinemia and is seen in approximately 60% of term infants and 80% of preterm infants [1,2,3]. However, severe hyperbilirubinemia can cause kernicterus, which is a serious condition that causes neurological problems [4, 5]. Although total serum bilirubin (TSB) measurement is the gold standard for evaluating of neonatal jaundice, it requires either venous blood sampling or capillary heel blood sampling, which involve invasive and painful procedures. Transcutaneous bilirubinometers use multiwavelength spectral reflectance from the skin surface to estimate the total serum or plasma bilirubin concentration. Studies have shown that the widespread use of transcutaneous bilirubin (TcB) or TSB measurements reduces the rate of rehospitalization for phototherapy (PT) among neonates with severe hyperbilirubinemia and lowers the proportion of infants receiving PT [6, 7]. However, because dermal bilirubin concentrations decrease faster than serum bilirubin, the transcutaneous bilirubinometer is less accurate during and after PT. Therefore, the measurement of TcB is not reliable in infants receiving PT [8]. However, a few studies have shown that TSB and TcB levels were correlated when TcB was measured from a part of the body covered with a radiopaque material during PT [9,10,11]. In a prospective observational study of 217 newborns, Costa-Posado et al. [12] evaluated the efficacy of indoor PT using a TcB measurement device. TSB and TcB measured from an area (sternum) covered by a radiopaque material were assessed at 24, 48, and 72 h of PT. They reported that TSB and TcB measurements were correlated in term and near-term neonates. Zecca et al. [13] compared the bilirubin levels of 364 preterm and term neonates using TSB and TcB values measured from skin that was covered during PT and determined that covered skin TcB correlated better with TSB than TcB measured from skin exposed to PT. Tan and Dong [8] reported that PT reduced but did not eliminate the correlation between TcB and TSB values.

In the present study, we aimed to evaluate the correlation between TSB and TcB measured from covered and exposed skin before PT, at 24th hour of PT, and 8 h after discontinuation of PT in both term and preterm neonates.

## Methods

Ethical approval was obtained from the hospital’s Clinical Research Ethics Committee (COA no. 669/2021). Written and verbal informed consent was obtained from all parents or legal guardians of the participating neonates.

This article includes a clinical photograph of a neonate. Written informed consent for publication of the image was obtained from the neonate’s parent before submission. The purpose, nature, and extent of publication, including electronic and open-access distributions, were fully explained. This work complies with the ethical standards outlined in the Declaration of Helsinki and adheres to the guidelines of the International Committee of Medical Journal Editors (ICMJE) regarding patient privacy and informed consent.

This prospective, single-center, case-controlled study was conducted in the Neonatal Intensive Care Unit (NICU) of a tertiary care hospital. The hospital averages approximately 1,500 births annually, and the level III NICU admits around 380 neonates/year.

### Study population

Between January and June 2022, 82 neonates diagnosed with indirect hyperbilirubinemia and requiring PT were assessed for eligibility. TcB measurements could not be performed in 8 neonates due to technical reasons, and consent was not obtained for 4 neonates. Ultimately, 70 neonates were enrolled in the final study cohort.

### Inclusion criteria

- Gestational age between 31 weeks and 42 weeks
- Postnatal age between 0 days and 28 days
- Diagnosis of indirect hyperbilirubinemia with an indication for PT
  ○ For infants ≥35 weeks gestational age: criteria based on the 2004 American Academy of Pediatrics (AAP) guidelines, including hour-specific bilirubin levels, gestational age, and risk factors [5]
  ○ For infants <35 weeks gestational age: criteria based on the 2018 Turkish Neonatology Association jaundice guidelines, utilizing weight-based nomograms adjusted for postnatal age [14]
- Parental/legal guardian consent

### Exclusion criteria

- Direct (conjugated) hyperbilirubinemia
- Major congenital anomalies
- Severe sepsis
- Hemodynamic instability
- Requirement for exchange transfusion
- Rehospitalization for recurrent jaundice

### Data collection

Comprehensive demographic and clinical data were recorded for all enrolled neonates. This included sex, gestational age at birth, birth weight, mode of delivery, age at hospital admission, length of hospital stay, maternal health conditions (e.g., preeclampsia, hypothyroidism, gestational diabetes), presence of pathological weight loss (>10% of birth weight), presence of cephalohematoma, maternal and neonatal blood groups, direct Coombs (DC) test results, and relevant laboratory values.

The present study aimed to evaluate whether covering the forehead with a radiopaque patch during the PT procedure increased the reliability of TcB measurement use in neonates receiving PT.

**Figure 1.**
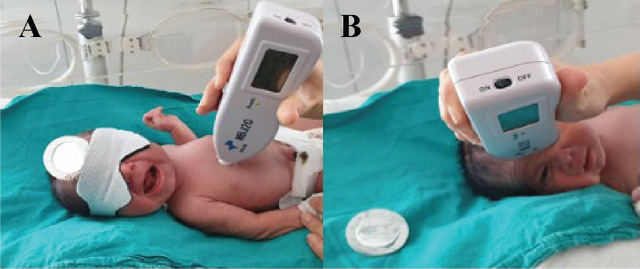
TcB measurements after PT. **(A)** TcB measurement at the exposed area and **(B)** TcB measurement under the covered shield. The neonate’s face is shown with full, explicit written informed consent obtained from the parents for publication of this identifiable image. The parents were informed about the nature, purpose, and extent of the publication, including digital distribution. PT, phototherapy; TcB, transcutaneous bilirubin.

In routine practice in our clinic, the eyes of newborns receiving PT are covered with PT goggles to prevent ocular exposure to PT light. The diaper area is also covered by a diaper. Apart from these areas, the entire body is exposed to PT. For this study, a 3 cm × 3 cm area of the forehead was covered with an aluminum-coated opaque patch (AirLife Temperature Probe Heat Reflective Patch, USA; **Figure 1**). Infants are turned from supine to prone position every 3 h during nursing care to ensure that all parts of the body receive PT.

TcB measurements were performed using an MBJ20-2019 Transcutaneous Handheld Bilirubinometer (Beijing M&B Electronic Instruments Co., Ltd., China, 2019). PT was administered using a Natus neoBLUE LED Phototherapy System (15 ± 2 µW/cm^2^/nm, 35 ± 3.5 µW/cm^2^/nm, 2008, Canada) and/or Bilisphere 360 LED Phototherapy System (Novos, Turkey).

At admission, each neonate underwent TcB measurement from the sternum and forehead, and the values were recorded together with the TSB value. TSB levels were determined from 2 cc blood samples in the biochemistry laboratory of our hospital. The samples were collected into BD Vacutainer tubes (Beckton Dickinson, New Jersey, USA), centrifuged at 4000 × *g* for 10 min, then measured spectrophotometrically after the diazo reaction using a biochemistry autoanalyzer (Roche Diagnostics Cobas C501 Model, Istanbul/Turkey). TSB and TcB levels from covered (forehead) and exposed (sternum) areas were analyzed at 24th hour of PT and 8 h after discontinuation of PT (**Figure 1**).

### Statistical analysis

Descriptive statistics were presented as mean, standard deviation (SD), and range for quantitative variables with normal distribution, median, and range for quantitative variables without normal distribution, and number and percentage for qualitative variables. Comparisons between two dependent groups were made using paired samples *t*-tests. *P* < 0.05 was accepted as statistically significant. Agreement between TSB levels and covered and exposed skin TcB levels was evaluated using the intraclass correlation coefficient (ICC) with 95% confidence intervals (CIs). The distribution of the cases was indicated by the Bland-Altman plot. SPSS version 11.5 software was used for statistical analyses.

## Results

This study was conducted with 70 neonates in need of PT on postnatal days 0–28. Among the 70 neonates, 36 (51.4%) were female. The mean birth weight was 3020 ± 556 g, and the median gestational age was 38 weeks (range: 31–41). Seventeen infants (24.3%) were preterm and 53 (75.7%) were term. Forty-one infants (58.5%) had ABO incompatibility, 3 (4.2%) had Rh incompatibility, and 1 (1.4%) had both ABO and Rh incompatibility. The DC test was positive in 23 infants (32.8%). The epidemiological characteristics of the neonates included in the study are presented in **Table 1**.

**Table 1.** Epidemiological characteristics of the neonates receiving PT for hyperbilirubinemia

**Characteristics**		**N (%)**
Gestational age (weeks), median (range)	38 (31–42)	
Birth weight (g), mean ± SD	3020 ± 556	
Sex		
Female		36 (51.4)
Male		34 (48.6)
Maternal age (years), mean ± SD	26.3 ± 5.7	
Maternal disease status		
Yes		8 (11.4)
No		60 (88.6)
Mode of delivery		
NSVD		51 (72.9)
C/S		19 (27.1)
Age at admission (days), median (range)	2 (1–13)	
Length of stay (days), median (range)	3 (2–27)	
Blood incompatibility		
None		25 (35.8)
ABO incompatibility		41 (58.6)
Rh incompatibility		3 (4.2)
ABO + Rh incompatibility		1 (1.4)
DC test		
Negative		47 (67.2)
Positive		23 (32.8)
Pathological weight loss		
Yes		10 (14.3)
No		60 (85.7)
Cephalic hematoma		
Yes		2 (2.9)
No		68 (97.1)

C/S, Cesarean section; DC, direct Coombs; NSVD, normal spontaneous vaginal delivery; PT, phototherapy; SD, standard deviation.

Comparisons of TSB and TcB measurements from covered and exposed skin performed before PT, at 24th hour of PT, and 8 h after discontinuation of PT are shown in **Table 2**. Before PT, the difference between mean TSB and covered skin TcB values was 0.79 mg/dL (*P* = 0.035; CI: 0.05–1.52), and the difference between mean TSB and exposed skin TcB values was 0.76 mg/dL (*P* = 0.004; CI: 0.03–1.50). There was a 0.02 mg/dL difference between the mean covered and exposed skin TcB values. At 24th hour of PT, the differences between TSB and covered skin TcB and between TSB and exposed skin TcB were 0.60 mg/dL (*P* = 0.026; CI: 0.07–1.13) and 3.53 mg/dL (*P* < 0.001; CI: 3.05–4.02), respectively. At 8 h after discontinuation of PT, these differences were 0.71 mg/dL (*P* = 0.006; CI: −1.21 to −0.20) and 1.04 mg/dL (*P* < 0.001; CI: 0.49–1.60), respectively.

**Table 2.** Comparison of TSB and TcB levels from covered and exposed skin measured before, during, and after PT

	**Mean ± SD**		
	**Before PT**	**At 24th hour of PT**	**8 h after discontinuation of PT**
TSB	11.91 ± 4.48	7.67 ± 2.34	7.90 ± 2.09
Covered skin TcB	11.11 ± 3.01	7.07 ± 2.25	8.61 ± 2.39
Exposed skin TcB	11.14 ± 3.11	4.13 ± 1.88	6.85 ± 2.44

PT, phototherapy; SD, standard deviation; TcB, transcutaneous bilirubin; TSB, total serum bilirubin.

Agreement between TSB and covered and exposed skin TcB values measured at each time point was evaluated using an ICC of 0.5–1 as an indicator of compatibility. ICC values closer to 1 indicate stronger agreement. The ICC values with 95% CIs for these comparisons are given in **Table 3**.

**Table 3.** Correlation between TSB values and TcB values measured from covered and exposed skin

	**Before PT ICC (95% CI)**	**After 24 h of PT ICC (95% CI)**	**8 h after PT ICC (95% CI)**
TSB vs. exposed skin TcB	0.667 (0.513–0.780)	0.229 (0.092–0.544)	0.432 (0.200–0.613)
TSB vs. covered skin TcB	0.665 (0.508–0.778)	0.520 (0.326–0.672)	0.537 (0.339–0.687)
Exposed vs. covered skin TcB	0.828 (0.737–0.890)	<0.245 (0.095–0.542)	0.557 (0.056–0.784)

CI, confidence interval; ICC, intraclass correlation coefficient; PT, phototherapy; TcB, transcutaneous bilirubin; TSB, total serum bilirubin.

**Figure 2.**
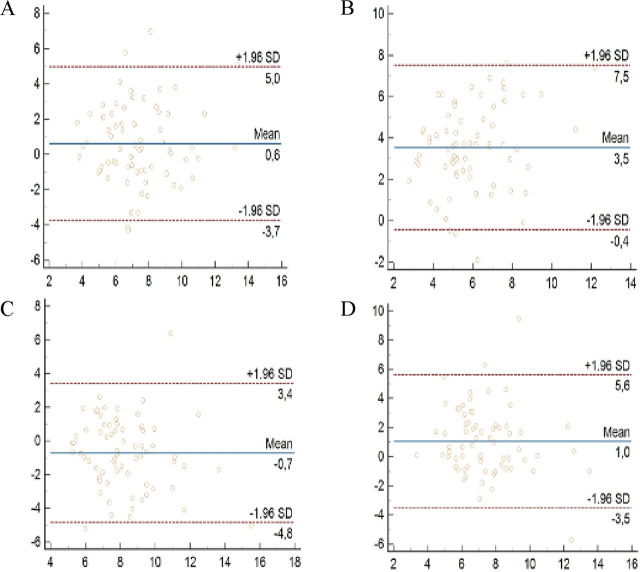
Correlation between TSB and covered skin TcB **(A)** and exposed skin TcB **(B)** measurement after 24 h of PT. Correlation between TSB and covered skin TcB **(C)** and exposed skin TcB **(D)** measurements 8 h after discontinuing PT. The Bland-Altman plot shows the average of the two methods on the X-axis and the absolute difference between the two methods on the Y-axis. PT, phototherapy; TcB, transcutaneous bilirubin; TSB, total serum bilirubin.

Bland-Altman scatterplots showing the correlations between TSB and covered skin TcB values and between TSB and exposed skin TcB values at 24th hour of PT and 8 h after discontinuation of PT are shown in **Figure 2**.

## Discussion

The present study aimed to evaluate the compatibility of TcB measurements with TSB values in neonates undergoing PT for indirect hyperbilirubinemia. During PT, a radiopaque patch was applied to the forehead to shield a specific area from light exposure. TcB measurements were obtained from both the covered and exposed skin areas, and TSB levels were measured simultaneously at three time points: before initiation of PT, at the 24th hour of PT, and 8 h after discontinuation of PT.

Although the gold standard for diagnosing hyperbilirubinemia remains the quantification of TSB from venous blood samples, this method is invasive, painful, and often distressing for neonates. As a result, several non-invasive devices capable of estimating bilirubin levels transcutaneously have been developed and are now widely utilized in clinical practice. These devices offer a safer and more comfortable alternative for monitoring bilirubin levels, particularly when used under appropriate conditions, such as on skin shielded from PT exposure. Thus, the use of TcB measurement has been included in guidelines as a screening method for neonatal hyperbilirubinemia [14]. Transcutaneous bilirubinometers are of practical use because of their suitability for daily use, both in and out of the hospital, and their ability to non-invasively measure total bilirubin from the skin surface [8, 11]. However, the effectiveness of TcB measurement varies due to factors such as race, skin color, and body hair growth [15, 16].

Before 30 weeks of gestation, the neonatal epidermis is notably thin, consisting of only a few cellular layers and an underdeveloped stratum corneum. However, by approximately 34 weeks, the epidermis undergoes substantial maturation. In preterm infants, postnatal adaptations significantly accelerate epidermal development, such that by the end of the second postnatal week, even the most immature neonates exhibit epidermal histology similar to that of term infants. A TcB device measures the optical density difference between blue and green light wavelengths, minimizing the effects of melatonin pigment and skin maturity [12].

In addition, there are studies showing that PT negatively affects the correlation between TcB and TSB measurements [8, 17, 18]. PT works by the principle of isomerization and clearance of cutaneous bilirubin. As a transcutaneous bilirubinometer measures cutaneous bilirubin, TcB measurements may not yield accurate results in patients receiving PT. In our clinic, TSB measurements are used to make PT decisions and in post-treatment evaluations. However, TSB monitoring requires frequent blood collection from infants. Moreover, it is known that the jaundice-reducing effect of light exposure occurs only in exposed areas, while unexposed areas remain icteric [13]. The present study was designed to evaluate the accuracy of TcB measurements from areas of skin exposed and unexposed during PT to reduce blood sampling for TSB measurement in neonates.

In a study of 39 patients by Fonseca et al. [9] examining the reliability of TcB values measured from covered skin during and after PT, the mean TSB level was reported as 11.5 ± 2.7 mg/dL and the TcB level as 11.1 ± 2.7 mg/dL before PT. In the present study, TSB and covered skin TcB (forehead) values measured before PT were 11.91 ± 4.48 mg/dL and 11.11 ± 3.01 mg/dL, respectively (**Table 2**). These results indicate that TcB measurements can be used reliably before PT is initiated.

In another study with 364 patients, Zecca et al. [13] compared covered skin TcB and TSB levels in preterm and term neonates during PT. According to their study, TSB was found to be 9.2 ± 3.3 mg/dL and TcB was 9.0 ± 3.6 mg/dL, and the mean difference was 0.2 mg/dL. In the present study, at 24th hour of PT, TSB was measured as 7.67 ± 2.34 mg/dL and TcB was 7.07 ± 2.25 mg/dL, and the mean difference was 0.6 mg/dL (**Table 2**). These results show that TcB measured from a covered area can be used reliably during PT.

A prospective cohort study by Murli et al. [19] evaluated the reliability of TcB measured from covered skin in 100 newborns receiving PT. Measurements were made before starting PT, after 12 h and 24 h of PT, and 12 h after discontinuing PT. At 12 h after PT, TSB was 11.3 ± 2.7 mg/dL and TcB measured from covered skin was 13.1 ± 2.8 mg/dL. In the present study, measurements were made at 8 h after PT and TSB was found to be 7.90 ± 2.09 mg/dL, covered skin TcB was 8.61 ± 2.39 mg/dL, and exposed skin TcB was 6.85 ± 2.44 mg/dL (**Table 2**). While the covered skin TcB values were close to the TSB level at 8 h after discontinuation of PT, the significant difference between TSB and exposed skin TcB levels persisted. These results show that even at 8 h after discontinuation of PT, TcB measurements from covered areas can still be used reliably.

Cat et al. [1] evaluated the relationship between TcB and TSB in 105 newborns with jaundice and reported a correlation of 0.850 before PT. Pratesi et al. [20] compared the Bilicare and Minolta JM-103 Bilicheck transcutaneous bilirubinometers in 458 late preterm and term newborns and reported a correlation coefficient of 0.55 between TSB and TcB measured with the Bilicheck before PT. In another study of 78 newborns, Khajehei et al. [21] compared TcB measurement and TSB levels and reported their correlation coefficient to be 0.805 before PT. In the present study, we determined an ICC of 0.665 between TSB and TcB measured with the MBJ20-2019 Transcutaneous Handheld Bilirubinometer (Beijing M&B Electronic Instruments Co., Ltd, China, 2019) before PT. The correlation coefficient being >0.5 suggests that the device used in our unit is reliable in the measurement of bilirubin. However, based on the study of Khajehei et al. [21], the lower correlation may be due to the use of different devices.

Esmaelzadeh-Saeieh et al. [22] investigated the relationship between TcB, TSB, and bilirubin levels measured using the Kramer Scale method in 180 newborns. The correlation between TSB and TcB from covered skin after PT was found to be 0.604. Varughese [10] investigated the reliability of TcB from covered skin measured after 4, 12, and 24 h of PT in 450 newborns. They covered an area (sternum) with an opaque cover and reported a correlation of 0.869 for TSB and TcB measurements obtained from the covered area with the JM-105 TcB device at 24th hour of PT. In the present study, the ICC for TcB and TSB measured from covered skin at 24th hour of PT was 0.520 (**Table 3**). The relatively low agreement between these values in the present study may be due to the lower sample size and the use of different devices.

In a study of dermal bilirubin kinetics during PT in 33 term neonates, Özkan et al. [23] measured TSB and covered skin TcB before and after 6, 12, 18, 30, 42, and 66 h of PT. The correlation between TSB and covered skin TcB values measured after PT was found to be 0.500. In the present study, the ICC between covered skin TcB and TSB at 8 h after discontinuation of PT was 0.537 (**Table 3**).

We also evaluated the correlation between TcB measurements and TSB at values above and below a threshold of 12 mg/dL. In patients with TSB concentrations >12 mg/dL, there was a low correlation between TSB level and TcB measurements before and immediately after PT. This supports that TcB measurements are more reliable at values of 12 mg/dL and lower.

Some limitations of the present study are that it was a single center, included a limited sample, and measurements were obtained with a single TcB device. Subgroup analyses could not be performed due to the small number of preterm neonates. Moreover, measurements could not be performed at 24 h and 48 h after PT because most of the patients were discharged. Therefore, it was not possible to obtain information about whether the difference in correlation of TSB with TcB from covered and exposed skin disappeared later after discontinuing PT. In addition, although in our study design, we intended to include all newborns who received PT, some could not be included in the study for various reasons.

## Conclusion

The findings of the present study suggest a close correlation between TcB levels measured from covered skin and TSB measurements in newborns. Therefore, TcB measurements from a covered area could serve as a reliable alternative to blood collection in neonates undergoing PT for jaundice. These results have important implications for clinical practice, as non-invasive transcutaneous measurements can minimize the need for blood sampling, reduce patient discomfort, and potentially streamline the monitoring process for neonatal jaundice.
